# Localized abdominal wall metastasis of papillary renal cell carcinoma: a case report

**DOI:** 10.3389/fsurg.2024.1413188

**Published:** 2024-11-08

**Authors:** Chadi Nahal, Claire Wunker, Jennifer Keller

**Affiliations:** Department of Surgery, Saint Louis University School of Medicine, St. Louis, MO, United States

**Keywords:** papillary, renal cell carcinoma, cutaneous metastasis, localized, abdominal wall reconstruction

## Abstract

**Introduction:**

Papillary renal cell carcinoma accounts for one tenth of all renal cell carcinomas. Compared to other renal cell carcinoma subtypes, it is more often localized at the time of diagnosis and rarely metastasizes to the skin. There are no previously reported cases of cutaneous papillary renal cell carcinoma localized to the abdominal wall which we present here.

**Case presentation:**

A 77 year-old female with multiple previous cancers, including a stage 1 left papillary renal cell carcinoma, treated with partial nephrectomy 32 months prior to presentation, was found to have a left upper abdominal wall mass on interval screening computed tomography. Fine needle aspiration was performed, obtaining limited tissue, followed by incisional biopsy. Histology and immunohistochemistry were consistent with renal cell carcinoma. She underwent operative excision of the abdominal wall mass with reconstruction using mesh and left posterior rectus fascial release. Histology and immunohistochemistry of the operative specimen reconfirmed the diagnosis of cutaneous metastasis of renal cell carcinoma. She was treated with adjuvant pembrolizumab and has no existing evidence of disease.

**Conclusions:**

Papillary renal cell carcinoma metastasized to the skin is uncommon, especially when localized to the abdominal wall without any other sites of metastases. Metastasis should be on the differential diagnosis when evaluating newly identified abdominal masses in patients with a history of papillary renal cell carcinoma. When localized, abdominal wall metastasis of papillary renal cell carcinoma can be effectively treated with resection and reconstruction, followed by systemic therapy when indicated.

## Background

Papillary renal cell carcinoma (pRCC) comprises 10%–15% of cases of renal cell carcinoma (RCC) (1). It is nearly twice as common in males with a threefold increased risk amongst African Americans. Additional associated risk factors for pRCC include obesity, hypertension, smoking, and, unique to this subtype of RCC, a positive correlation with chronic kidney disease stage (2).

pRCC is more likely to present localized to the kidney than the clear cell subtype (cRCC) (74.9% vs. 62.9%) (3) and can be treated with partial or radical nephrectomy (4). Despite the higher rate of localized disease at diagnosis, pRCC has worse local and distant recurrence free survival following partial nephrectomy than cRCC at 10 years (73% vs. 96.1%), despite being similar at 5 years (95.6% pRCC vs. 98.7% cRCC (5). Partial nephrectomy can be beneficial in maintaining kidney function in patients with tumors less than 10 cm in diameter and has similar oncologic control (6). A similar trend for worse survival in pRCC compared to cRCC is seen in metastatic disease (7).

On computed tomography (CT) imaging, pRCC appear homogenous, solid, and less vascular compared to other types of RCC, as indicated by decreased contrast enhancement relative to the renal cortex (8). 25% of lesions can have cystic changes (9) (Honda et al.), and metastatic lesions have similar imaging characteristics to the primary tumor (Vikram et al.) (8). pRCC presents with metastasis in 9.6% of patients (3).

To our knowledge, there are only 4 other reported cases of pRCC with metastasis to the skin (10, 11). We present the case of a 77-year-old female who presents with a cutaneous abdominal wall metastasis of pRCC nearly 3 years following partial nephrectomy of her primary tumor.

## Case presentation

**Figure 1:**
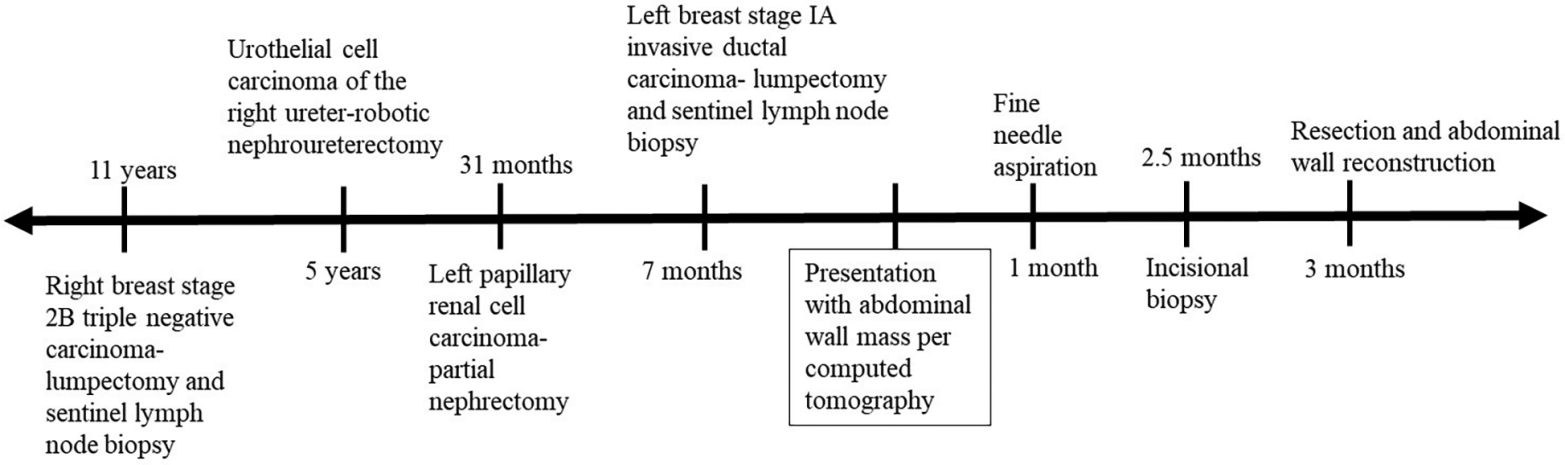
The patient's oncologic history shown before and after her computed tomography scan indicated a newly identified abdominal wall mass.

Our patient is a 77-year-old Caucasian female with past medical history significant for multiple previous cancers: including a stage 1 left pRCC status post robotic partial nephrectomy 32 months prior to presentation, a stage 0 urothelial carcinoma of the right ureter (status post robotic nephroureterectomy 5 years prior to presentation), a stage IA left breast invasive ductal carcinoma (status post lumpectomy and sentinel lymph node biopsy with post-operative anastrozole 7 months prior to presentation), and a stage 2B right breast triple negative carcinoma (status post lumpectomy and sentinel lymph node biopsy, chemotherapy and radiation 11 years prior to presentation) who presented with a left upper quadrant abdominal wall mass. A timeline of her oncologic history is illustrated in Figure 1.

She had a hysterectomy and bilateral salpingoophorectomies for a benign lesion approximately 20 years prior and a parathyroidectomy for an adenoma 18 years prior to presentation. She underwent genetic testing for mutations predisposing for malignancy which was negative. Family history was notable for breast cancer in her mother and in a maternal aunt, lung cancer in a second maternal aunt, Hodgkin's lymphoma in a maternal cousin, liver cancer (unspecified) in the patient's father, and neural tube defects in the patient's son (died at age 14). She has 10 pack-year smoking history but quit 5 years prior to presentation.

She was undergoing routine surveillance CT imaging for her pRCC and a new left upper quadrant abdominal wall mass was noted. It showed a multi-lobulated, ovoid soft tissue mass up to 2.5 cm in diameter with subcutaneous soft tissue and peritoneal involvement. Per physical examination, the subcutaneous tissue was distant from any visible incisional scars or previous port sites.

The patient underwent ultrasound guided needle aspiration of the skin lesion, which resulted in the mass being no longer identifiable on ultrasound following aspiration. Immunohistochemical stains were positive for CAM5.2, PAX8 and AMACR, and negative for GATA-3, CK 7 and CA IX. The specimen had limited cellularity and bland epithelium, insufficient for a definitive diagnosis; however, it had similar histology and immunohistochemistry to the primary pRCC. Therefore, we proceeded with excisional biopsy of the left upper quadrant abdominal wall. This was done operatively under general anesthesia for multiple reasons: it was no longer palpable, it was not visible under ultrasound following fine needle aspiration, and the possibility for abdominal wall reconstruction vs. mesh placement after removal of the mass.

Histological analysis of the operative incisional biopsy specimen showed multiple cysts lined by a single layer of columnar epithelium with abundant eosinophilic cytoplasm and round nuclei with occasional prominent nucleoli. This was similar to the histology of the previously resected primary renal lesion. Immunostaining of the tumor cells was consistent with the needle aspiration, and negative for additional markers tested, including calretinin, CK20, pankeratin, EMA, napsin-A, TTF1, c-Kit, CDX2 and HMB-45. The morphologic and immunophenotypic features supported diagnosis of an epithelial neoplasm compatible with renal origin given the reactivity for PAX8

The specimen was sent for further testing at the Mayo Clinic Laboratories (3050 Superior Drive NW, Rochester, MN 55901), and the tumor cells were positive for TFE3 by immunostaining; however, TFE3 fluorescence *in situ* hybridization testing was negative for rearrangement. Immunostains showed retained cytoplasmic reactivity for FH and SDHB in the tumor cells, with weak reactivity for 2SC.  These findings argued against classification as FH-deficient or SDH-deficient renal cell carcinoma.  The biopsied neoplasm was classified as renal cell carcinoma, not otherwise specified.

Because the lesion was localized to the abdominal wall, including subcutaneous and peritoneal tissue without any other sites of distant metastasis, the patient underwent excision of the abdominal wall mass with reconstruction using mesh and release of left posterior rectus fascia. There was involvement of multiple layers within the abdominal wall. These were resected using electrocautery, including involved rectus muscle, subcutaneous tissue, and a nodule adherent but separate from the posterior rectus fascia. Once this was done, the posterior rectus sheath was mobilized to approximate the abdominal wall. The defect was roughly 10 cm × 15 cm. A 16 cm × 24 cm absorbable mesh was then placed in an underlay position and secured with interrupted PDS sutures. The abdominal wall was closed using looped polydioxanone in standard fashion. With the release of the posterior fascia the abdomen came together without significant tension. A drain was placed over the closure in the subcutaneous defect.

The post-operative course was uncomplicated and she was discharged 4 days post-operatively in stable condition with adequate pain control. Histology and immunohistochemistry of the resected specimen was consistent with the findings of the excisional biopsy and her known RCC. She started adjuvant pembrolizumab for 1 year.

## Discussion

We present a rare case of delayed localized cutaneous metastasis of a stage 1 pRCC. Cutaneous metastasis of RCC is uncommon. One study following 306 cases of RCC over 12 years, which were either metastatic or non-metastatic at presentation, found a 3.3% prevalence of cutaneous metastasis, with the scalp being the most common site, followed by thoracic and abdominal sites (12). After cutaneous involvement is detected, mean survival was 7 months. A systematic review showed a similar incidence of cutaneous metastasis at 3.3% in RCC presenting at a variety of stages, with survival averaging 10.9 months from diagnosis of cutaneous metastasis (13). The more typical sites of RCC metastasis, from highest to lowest frequency, include lung (45%), bone (30%), lymph nodes (22%), liver (20%), and brain (8%) (14). Cutaneous metastasis is even more rare for the papillary subtype, with only 4 other reports in the literature. In RCC cutaneous metastasis can be via lymphatic or hematogenous routes, but also from surgical site seeding or direct neoplastic invasion (15). It most frequently appears nodular, with color akin to skin, but can also be erythematous or have a purplish hue given the highly vascularized nature of RCC (15). The previously published cases of cutaneous pRCC vary in regard to the site of metastasis and initial presentation of pRCC, but none had localized abdominal wall metastasis like our patient.

Of the previously published cases, two had metastatic pRCC at diagnosis. One of these patients was diagnosed with pRCC 3 months prior to symptoms of cutaneous metastasis. She then presented with breast and chest erythematous patches and plaques, along with peau d’orange and telangiectasias that appeared despite being on temsirolimus. She received symptomatic treatment and continued temsirolimus therapy but died 2 months following presentation of skin symptoms (16). Another case of pRCC metastatic to the lung and bone presented with skin nodules on the upper extremities and torso (17). The patient died 6 months after starting temsirolimus.

Two other cases had localized pRCC at the time of diagnosis. One had localized type 1 pRCC, but was a poor surgical candidate for nephrectomy. The patient was treated with sunitinib and developed metastasis to bone, lung, and brain. 4 years after diagnosis, he presented with multiple skin nodules on the trunk and proximal extremities (10). The other case involved pRCC treated with nephrectomy 7 years prior to presentation who developed a scalp nodule that was excised and recurred in 12 months and was then diagnosed as metastatic pRCC (11). Our patient was treated with adjuvant pembrolizumab, which has been shown in a single-arm study to have an objective response rate of 26.7% in non-clear cell subtypes (18). Generally, the various subtypes of renal cell carcinoma, when metastasized in a cutaneous manner, can be resected if there is a single local lesion (19). Radiotherapy and systematic are also used for unresectable or disseminated lesions.

The immunohistochemical staining profile of our patient's skin biopsy had unusual features compared to the other cases of cutaneous pRCC. The immunohistology of abdominal wall lesion in our patient was negative for most typical stains of pRCC. These include napsin-A, GATA3, CK7, CK20, EMA, c-KIT, and CAIX (1, 20, 21). The diagnosis was established based on histology consistent with the patient's clinical history of type 2 pRCC, along with positive immunohistochemical staining of PAX8 and AMACR. TFE3 positivity without translocation can also be a surrogate marker for pRCC and suggests a poorer prognosis (22). Therefore, knowledge of this patient's history of localized pRCC treated with nephrectomy was crucial to diagnosing her abdominal wall lesions as metastatic pRCC.

## Conclusion

We present the first case in the literature of cutaneous metastasis of pRCC to a localized region of the abdominal wall. Patients presenting with a cutaneous abdominal wall mass should have a thorough assessment of their oncologic history with consideration of metastatic disease in the diagnostic differential. Cutaneous metastasis of pRCC is rare and can present with a vague immunohistochemical profile that requires consideration of both oncological history and histology for diagnosis.

## Data Availability

The original contributions presented in the study are included in the article/Supplementary Material, further inquiries can be directed to the corresponding author.
